# Supplementary material for: Development and Biochemical Characterization of Self-Immolative Linker Containing GnRH-III-Drug Conjugates
Source: Int J Mol Sci. 2022 May 3;23(9):5071. doi: 10.3390/ijms23095071 (PMC9105102; doi:10.3390/ijms23095071)
Supplement: Supplementary file 1 [file ijms-23-05071-s001.zip › ijms-1693866-supplementary.pdf]

International Journal of *Molecular Sciences*

Supporting Information

Development and biochemical characterization of self-immolative linker containing GnRH-III-drug conjugates

Sabine Schuster [1,2], Éva Juhász [3], Gábor Halmos [4], Ines Neundorf [5], Cesare Gennari [6] and Gábor Mező [1,2,*]

[1] Faculty of Science, Institute of Chemistry, Eötvös Loránd University, 1117 Budapest, Hungary; sabine.schuster83@gmail.com
[2] ELKH-ELTE Research Group of Peptide Chemistry, Faculty of Science, Eötvös Loránd University, 1117 Budapest, Hungary
[3] Department of Pediatrics, Faculty of Medicine, University of Debrecen, 4032 Debrecen, Hungary; juhasze@med.unideb.hu
[4] Department of Biopharmacy, Faculty of Pharmacy, University of Debrecen, 4032 Debrecen, Hungary; halmos.gabor@pharm.unideb.hu
[5] Department of Chemistry, Institute of Biochemistry, University of Cologne, 50674 Cologne, Germany; ines.neundorf@uni-koeln.de
[6] Dipartimento di Chimica, Università degli Studi di Milano, 20133 Milano, Italy; cesare.gennari@unimi.it
* Correspondence: gabor.mezo@ttk.elte.hu ; Tel.: +36-1-372-2500
† Work was conducted at the Eötvös Loránd University , and while visiting the Universities of Cologne and Milan.
‡ Current address: Department of Pharmaceutical Biology/Pharmacognosy, Institute of Pharmacy, University of Halle-Wittenberg, 06120 Halle (Saale), Germany.

Table of content

Reagents and general procedures 3

NMR data and spetra

Fmoc-Val-NHS 4

Fmoc-Val-Cit-OH 4

Fmoc-Val-Ala-OH 5

Fmoc-Val-Cit-PAB-OH 5

Fmoc-Val-Ala-PAB-OH 6

Fmoc-Val-Cit-PAB-Pnp 6

Fmoc-Val-Ala-PAB-Pnp 7

Table S1: Conjugation reaction of drug-linker and GnRH-III peptide 8

RP-HPLC profile and ESI-ion trap mass spectra of

*GnRH-III-[²His-³Trp,⁸Lys(glutaryl-Val-Cit-PABC-Dau)] (**10**)* 9

*GnRH-III-[²ΔHis-³D-Tic,⁸Lys(glutaryl-Val-Cit-PABC-Dau)] conjugate (**11**)* 9

*GnRH-III-[²His-³Trp,⁸Lys(glutaryl-Val-Ala-PABC-Dau)] conjugate (**12**)* 9

*GnRH-III-[²ΔHis-³D-Tic, ⁸Lys(glutaryl-Val-Ala-PABC-Dau conjugate (**13**)* 10

*GnRH-III-[²His-³Trp,⁸Lys(glutaryl-Val-Cit-PABC-diamine-PTX)] conjugate (**14**)* 10

*GnRH-III-[²ΔHis-³D-Tic,⁸Lys(glutaryl-Val-Cit-PABC-diamine-PTX)] conjugate (**15**)* 10

*GnRH-III-[²ΔHis-³D-Tic, ⁸Lys(glutaryl-Val-Ala-PABC-diamine-PTX)] conjugate (**16**)* 11

*GnRH-III-[²ΔHis-³D-Tic, ⁸Lys(glutaryl-Val-Ala-PABC-diamine-PTX)]conjugate (**17**)* 11

*GnRH-III-[²His-³Trp,⁸Lys(glutaryl-Dau)] conjugate (**23**)* 11

*GnRH-III-[²ΔHis-³D-Tic,⁸Lys(glutaryl-Dau)] conjugate (**24**)* 12

*GnRH-III-[²His-³Trp,⁸Lys(glutaryl-diamine-PTX)] conjugate (**25**)* 12

*GnRH-III-[²ΔHis-³D-Tic, ⁸Lys(glutaryl-diamine-PTX)] conjugate (**26**)* 12

Table S2: Chemical characteristics of the GnRH-III-Dau and -PTX bioconjugates 13

Western blot analysis of A2780 and Panc-1 cancer cells 13

Lysosomal degradation of in presence of rat liver lysosomal homogenate 14

Reagents and general procedures

Chemical Reagents

Amino acid derivatives and Rink-Amide MBHA resin were obtained from Iris Biotech GmbH (Marktredwitz, Germany), Novabiochem®/Merck-Millipore (Darmstadt, Germany) and Bachem (Bubendorf, Switzerland). Boc-aminooxyacetic acid (Boc-Aoa-OH), aminooxyacetic acid, scavengers, coupling agents (1-hydroxybenzotriazole hydrate (HOBt), *N,N′*-diisopropylcarbodiimide (DIC)), and cleavage reagents (triisopropylsilane (TIS), piperidine, 1,8-diazabicyclo[5.4.0]undec-7-ene (DBU), trifluoroacetic acid (TFA)), diisopropylethylamine (DIPEA), methanol (MeOH) and solvent for RP-HPLC acetonitrile (ACN) were purchased from Sigma-Aldrich Kft (Budapest, Hungary). Daunorubicin hydrochloride was provided from IVAX (Budapest, Hungary). *N,N*-dimethylformamide (DMF), dichloromethane (DCM) and diethyl ether (Et_2O) were purchased from Molar Chemicals Kft (Budapest, Hungary).

Analytical RP-HLPC

A Knauer 2501 HPLC system was used to prove the purity of the compounds. As a stationary phase a Macherey-Nagel Nucleosil C18 column (100 Å, 5µm, 250 mm x 4.6 mm) was used. A linear gradient elution (0 min 0% B; 5 min 0% B; 30 min 90%, 31 min 100%, 36 min 100%) was used at a flow rate of 1 mL/min with eluent A (0.1% TFA in water) and eluent B (0.1% TFA in ACN/H_2O (80:20, v/v)). Peaks were detected at 220 nm.

Mass spectrometry

Electrospray ionization (ESI) mass spectrometric analyses were performed on an Esquire 3000+ ion trap mass spectrometer (Bruker Daltonics, Bremen, Germany). Spectra were acquired in the 50–2500 *m/z* range. Samples were dissolved in a mixture of ACN/water (1:1, v/v) and 0.1% formic acid.

Liquid chromatography-mass spectrometry (System I) was carried out on the same ESI mass spectrometer used with an Agilent 1100 HPLC system and a diode array detector (Agilent, Waldbronn, Germany). A Supelco C18 column (3 µm, 2.1 x 150 mm) (Hesperia, CA, USA) was used with a linear gradient from 2–70% B in 25 min (eluent A: H_2O + 0.1% HCOOH; eluent B: ACN/H_2O (80:20) + 0.1% HCOOH at a flow rate of 0.2 mL/min) to separate the peptides. Spectra were recorded in positive ion mode in the 100–2500 *m/z* range.

Furthermore, LC-MS analysis were performed on a Q Exactive Focus, high resolution and high mass accuracy, hybrid quadrupole-orbitrap mass spectrometer (Thermo Fisher Scientific, Bremen, Germany) using online UHPLC coupling (System II). Separation was performed on a Waters C4 column (ACQUITY UPLC ® BEH300, 1.7 µm, 2.1 x 150 mm). A linear gradient elution (0 min 2%B, 1 min 2%B, 17 min 90%B, 17.5 min 100%B, 20 min 100%B) was used at a flowrate of 0.3 ml/min with eluent A (H_2O + 0.1% HCOOH) and eluent B (ACN/H_2O (80:20) + 0.1% HCOOH). Spectra were recorded in positive ion mode in the 200–2000 *m/z* range.

NMR-Data

Proton NMR spectra were recorded on a spectrometer operating at 400.16 MHz. Proton chemical shifts are reported in ppm (δ) with the solvent reference relative to tetramethylsilane (TMS) employed as the internal standard ($CDCl_3$ δ = 7.26 ppm; CD_2Cl_2, δ = 5.32 ppm; $[D]_6$ DMSO, δ = 2.50 ppm; CD_3OD, δ = 3.33 ppm). The following abbreviations are used to describe spin multiplicity: s = singlet, d = doublet, t = triplet, q = quartet, m = multiplet, bs = broad signal, dd = doublet of doublet.

Fmoc-Val-NHS (**1**)

^{1}H NMR (400 MHz, Chloroform-*d*) δ 7.75 (d, *J* = 7.6 Hz, 2H, $C^{ar}H$), 7.57 (d, *J* = 7.4 Hz, 2H, $C^{ar}H$), 7.38 (t, *J* = 7.5 Hz, 2H, $C^{ar}H$), 7.30 (t, *J* = 7.4 Hz, 2H, $C^{ar}H$), 4.67 (dd, *J* = 9.2, 4.6 Hz, 1H, $C^{\alpha}H$), 4.43 (d, *J* = 6.6 Hz, 2H, Fmoc-CH_2), 4.23 (t, *J* = 6.8 Hz, 1H, Fmoc-CH), 2.82 (d, *J* = 7.5 Hz, 4H, NHS-CH_2), 2.42 – 2.24 (m, 1H, $C^{\beta}H$), 1.04 (dd, *J* = 9.8, 7.0 Hz, 6H, $C^{\gamma}H_3$).

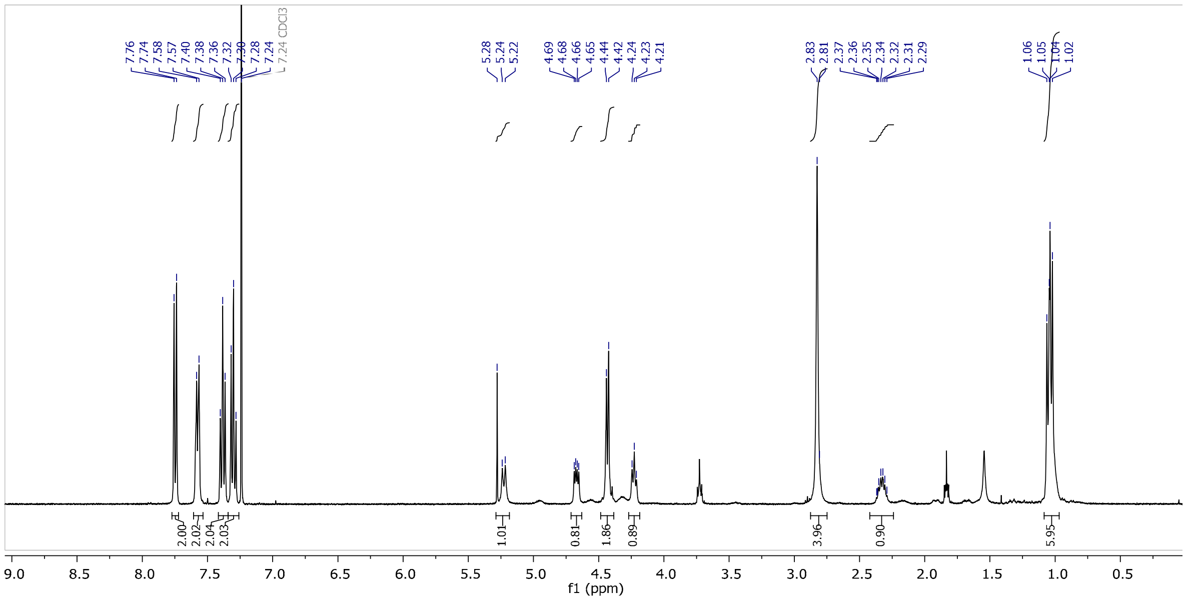

Fmoc-Val-Cit-OH (**2a**)

^{1}H NMR (400 MHz, Methanol-d_4) δ 7.80 (d, *J* = 7.5 Hz, 2H, $C^{ar}H$), 7.67 (t, *J* = 6.9 Hz, 2H, $C^{ar}H$), 7.39 (t, *J* = 7.4 Hz, 2H, $C^{ar}H$), 7.31 (t, *J* = 7.4 Hz, 2H, $C^{ar}H$), 4.43 – 4.32 (m, 3H, Fmoc-CH_2/Cit-$C^{\alpha}H$), 4.23 (t, *J* = 6.8 Hz, 1H, Val-$C^{\alpha}H$), 4.01 – 3.92 (m, 1H, Fmoc-CH), 3.10 (t, *J* = 6.7 Hz, 2H, Cit-CH_2), 2.13 – 1.99 (m, 1H, Val-$C^{\beta}H$), 1.97 – 1.84 (m, 1H, Cit-$C\underline{H^A}H^B$), 1.70 (m, 1H, Cit-$CH^A\underline{H^B}$), 1.60 – 1.49 (m, 2H,Cit-CH_2), 0.97 (dd, *J* = 11.4, 6.8 Hz, 6H, Val-$C^{\gamma}H_3$).

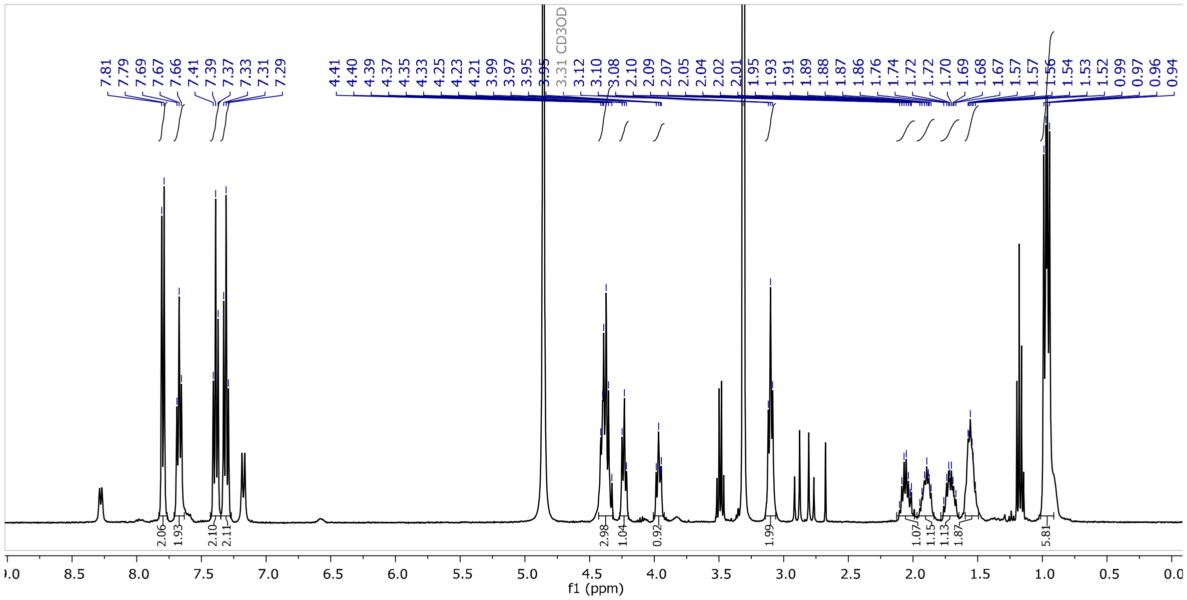

Fmoc-Val-Ala-OH (**2b**)

^{1}H NMR (400 MHz, Methanol-d_4) δ 7.79 (d, *J* = 7.6 Hz, 2H, $C^{ar}H$), 7.66 (t, *J* = 7.0 Hz, 2H, $C^{ar}H$), 7.38 (t, *J* = 7.5 Hz, 2H, $C^{ar}H$), 7.30 (t, *J* = 7.3 Hz, 2H, $C^{ar}H$), 4.43 – 4.31 (m, 3H, Fmoc-CH_2/ Ala-$C^{\alpha}H$), 4.26 – 4.19 (m, 1H, Val-$C^{\alpha}H$), 3.96 (t, *J* = 7.6 Hz, 1H, Fmoc-CH), 2.11 – 1.96 (m, 1H, Val-$C^{\beta}H$), 1.40 (d, *J* = 6.9 Hz, 3H, Ala-$C^{\beta}H_3$), 0.96 (dd, *J* = 15.5, 6.4 Hz, 6H, Val-$C^{\gamma}H_3$).

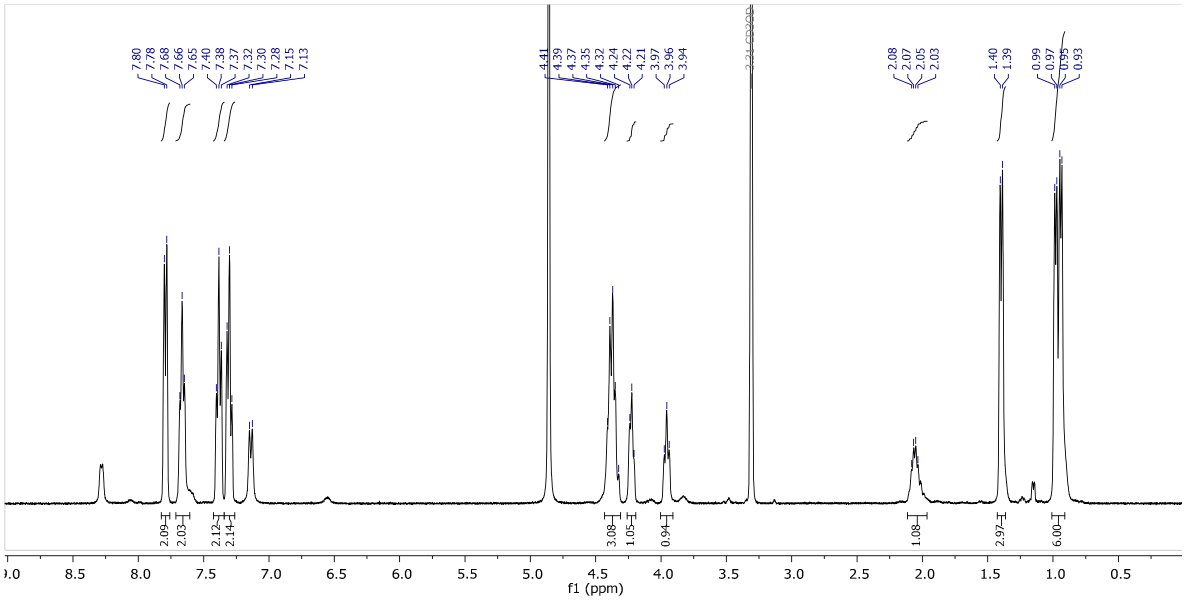

Fmoc-Val-Cit-PAB-OH (**3a**)

^{1}H NMR (400 MHz, DMSO-d_6) δ 9.97 (s, 1H, PAB-NH), 8.09 (d, *J* = 7.6 Hz, 1H, NH), 7.89 (d, *J* = 7.5 Hz, 2H, $C^{ar}H$), 7.74 (t, *J* = 7.8 Hz, 2H, $C^{ar}H$), 7.54 (d, *J* = 8.4 Hz, 2H, $C^{ar}H$), 7.40 (d, *J* = 9.6 Hz, 2H, $C^{ar}H$), 7.32 (t, *J* = 7.0 Hz, 2H $C^{ar}H$), 7.23 (d, *J* = 8.4 Hz, 2H, $C^{ar}H$), 5.99 – 5.94 (m, 1H, Cit-NH), 5.38 (d, *J* = 14.8 Hz, 2H, Cit-NH_2), 5.09 (t, *J* = 5.6 Hz, 1H, PAB-OH), 4.43 (d, *J* = 5.4 Hz, 3H, Fmoc-CH_2/Cit-$C^{\alpha}H$), 4.30 – 4.21 (m, 3H, PAB-CH_2/Val-$C^{\alpha}H$), 3.93 (dd, *J* = 9.1, 7.1 Hz, 1H, Fmoc-CH), 3.06 – 2.88 (m, 2H, Cit-NHCH_2), 2.06 – 1.91 (m, 1H, Val-$C^{\beta}H$), 1.76 – 1.64 (m, 1H, Cit-C$\underline{H^A}H^B$), 1.63 – 1.53 (m, 1H, Cit-C$H^A\underline{H^B}$), 1.48 – 1.33 (m, 2H, Cit-CH_2), 0.87 (dd, *J* = 10.8, 6.7 Hz, 6H, Val- $C^{\gamma}H_3$).

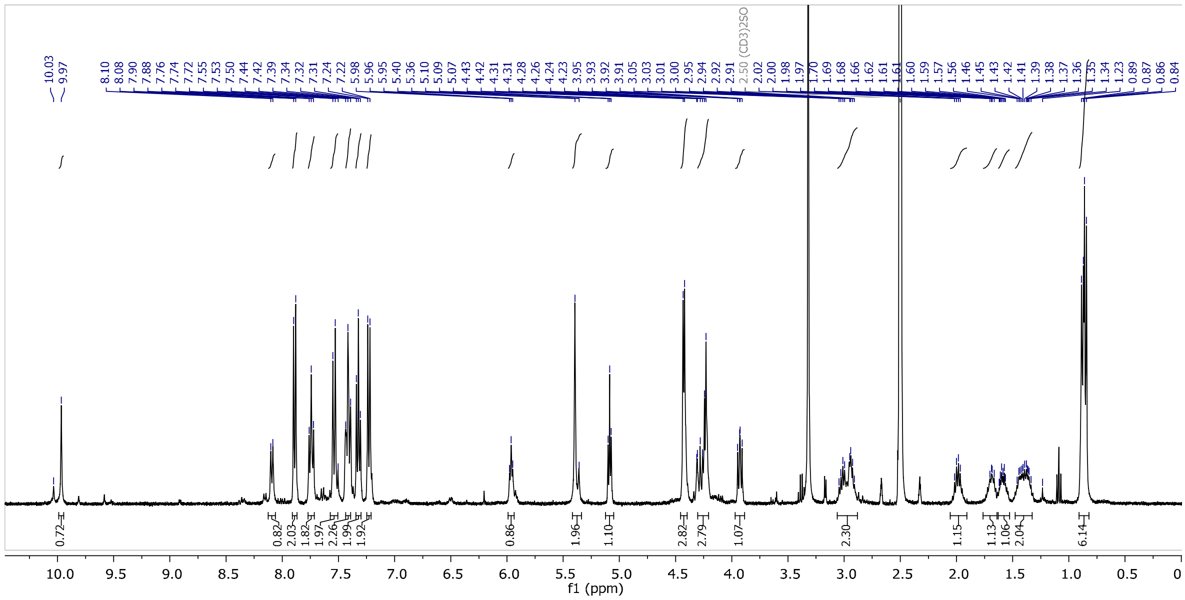

Fmoc-Val-Ala-PAB-OH (**3b**)

^{z1}H NMR (400 MHz, DMSO-d_6) δ 10.01 (s, 1H, PAB-NH), 8.16 (d, *J* = 7.0 Hz, 1H, NH), 7.88 (s, 2H, $C^{ar}H$), 7.74 (t, *J* = 7.2 Hz, 2H, $C^{ar}H$), 7.53 (d, *J* = 8.4 Hz, 2H, $C^{ar}H$), 7.43 – 7.39 (m, 2H, $C^{ar}H$), 7.32 (t, *J* = 7.4 Hz, 2H, $C^{ar}H$), 7.23 (d, *J* = 8.4 Hz, 2H, $C^{ar}H$), 5.09 (t, *J* = 5.7 Hz, 1H, PAB-OH), 4.43 (d, *J* = 5.6 Hz, 3H, Fmoc-CH_2/ Ala-$C^{\alpha}H$), 4.36 – 4.26 (m, 1H, Val-$C^{\alpha}H$), 4.23 (dd, *J* = 9.5, 2.9 Hz, 2H, PAB-CH_2), 3.95 – 3.87 (m, 1H, Fmoc-CH), 2.00 (dq, *J* = 13.4, 6.5 Hz, 1H, Val-$C^{\beta}H$), 1.30 (d, *J* = 7.1 Hz, 3H, Ala-$C^{\beta}H_3$), 0.88 (dd, *J* = 12.3, 6.8 Hz, 6H, Val-$C^{\gamma}H_3$).

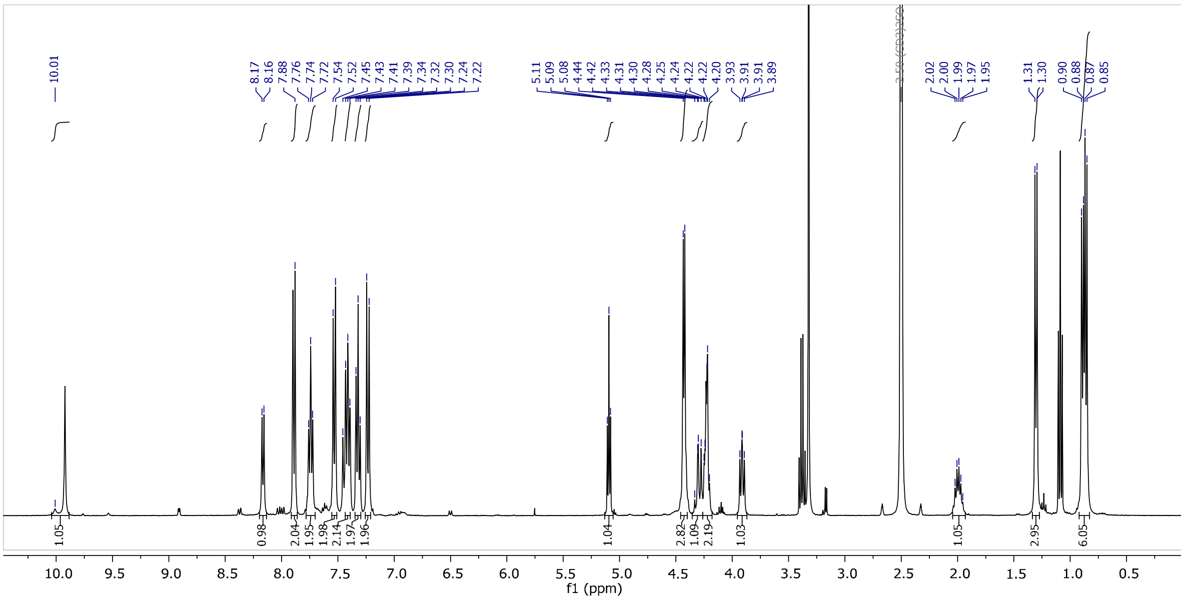

Fmoc-Val-Cit-PABC-Pnp (**4a**)

^{1}H NMR (400 MHz, DMSO-d_6) δ 10.14 (s, 1H, PAB-NH), 8.33 – 8.29 (m, 2H, $C^{ar}H$), 8.14 (d, *J* = 7.6 Hz, 1H, NH), 7.89 (d, *J* = 8.0 Hz, 2H, $C^{ar}H$), 7.74 (t, *J* = 7.6 Hz, 2H, $C^{ar}H$), 7.65 (d, *J* = 8.6 Hz, 2H, $C^{ar}H$), 7.59 – 7.55 (m, 2H, $C^{ar}H$), 7.41 (d, *J* = 8.4 Hz, 4H, $C^{ar}H$), 7.32 (td, *J* = 7.5, 1.1 Hz, 2H, $C^{ar}H$), 5.97 (t, *J* = 6.0 Hz, 1H, Cit-NH), 5.41 (s, 2H, Cit-NH_2), 5.25 (s, 2H, Fmoc-CH_2), 4.46 – 4.38 (m, 1H, Cit-$C^{\alpha}H$), 4.34 – 4.18 (m, 3H, PAB-CH_2/Val-$C^{\alpha}H$), 3.93 (dd, *J* = 8.9, 7.0 Hz, 1H, Fmoc-CH), 3.07 – 2.90 (m, 2H, Cit-$NHCH_2$), 2.03 – 1.94 (m, 1H, Val-$C^{\beta}H$), 1.76 – 1.65 (m, 1H, Cit-C<u>H^A</u>H^B), 1.61 (dd, *J* = 9.1, 4.8 Hz, 1H, Cit-CH^A<u>H^B</u>), 1.52 – 1.30 (m, 2H, Cit-CH_2), 0.87 (dd, *J* = 11.4, 6.9 Hz, 6H, Val- $C^{\gamma}H_3$).

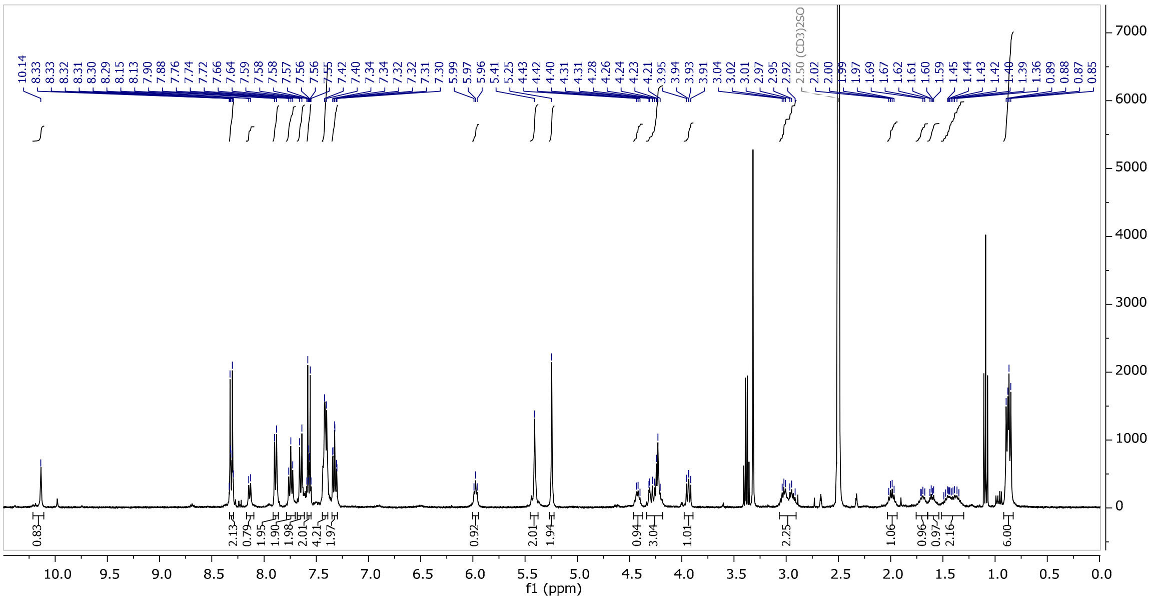

Fmoc-Val-Ala-PABC-Pnp (**4b**)

^{1}H NMR (400 MHz, DMSO-d_6) δ 10.09 (s, 1H, PAB-NH), 8.32 (s, 2H, C^{ar}H), 7.89 (d, *J* = 7.2 Hz, 2H, C^{ar}H), 7.80 – 7.70 (m, 2H, C^{ar}H), 7.64 (d, *J* = 8.8 Hz, 2H, C^{ar}H), 7.57 (d, *J* = 8.3 Hz, 2H, C^{ar}H), 7.42 (d, *J* = 7.8 Hz, 4H, C^{ar}H), 7.36 – 7.28 (m, 2H, C^{ar}H), 5.24 (s, 2H, Fmoc-CH_2), 4.49 – 4.37 (m, 1H, Ala-C^{α}H), 4.29 (d, *J* = 11.5 Hz, 1H, Val-C^{α}H), 4.22 (s, 2H, PAB-CH_2), 3.97 – 3.89 (m, 1H, Fmoc-CH), 2.04 – 1.94 (m, 1H, Val-C^{β}H), 1.31 (d, *J* = 5.8 Hz, 3H, Ala-$C^{\beta}H_3$), 0.88 (dd, *J* = 12.3, 6.4 Hz, 6H, Val-$C^{\gamma}H_3$).

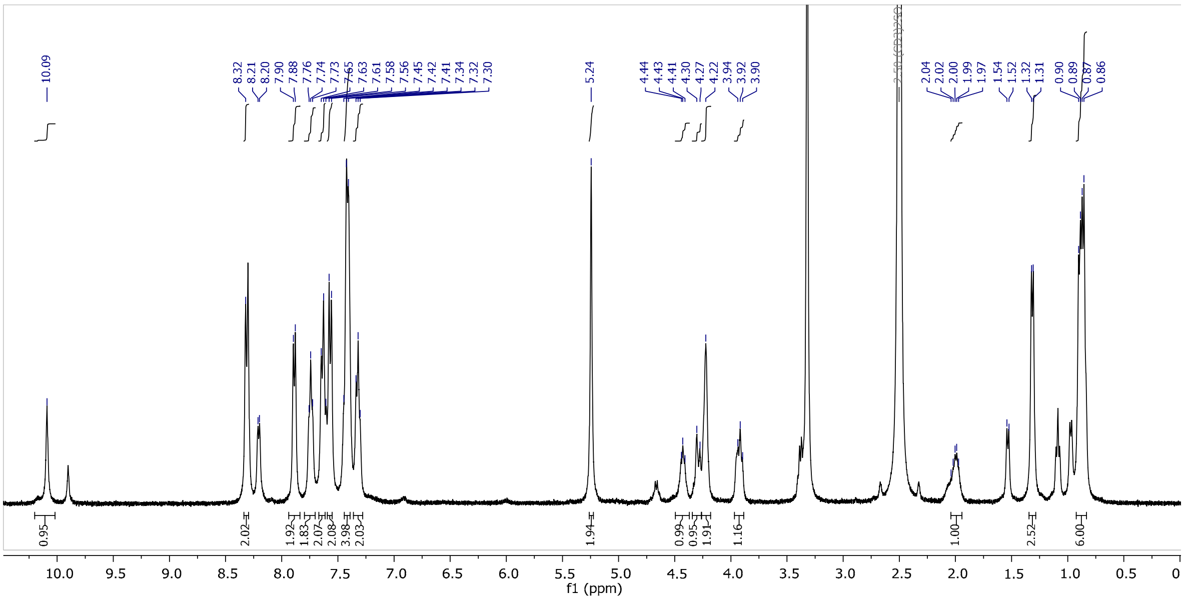

Conjugation reaction of drug-linker and GnRH-III peptide

Table S2: Ligation reaction. The linker (1 eq) was preactivated with HATU (0.9 eq) and DIPEA (2 eq) for 30 min, then peptide (1 eq) was added and reaction mixture was stirred for 24 hours.

Starting material							Product			
glutaryl-X-drug-Linker					Peptide (1 eq)		Conjugates			
Code	X	drug	[mg]	[µmol]	Code		Code	MW_{cal}	Yield	[mg]
**6a**	Val-Cit-PABC	Dau	3.3	3.10	**I**	[^{2}His-^{3}Trp]	**10**	2399.57	70%	3.9
**6a**	Val-Cit-PABC	Dau	5.0	4.78	**II**	[$^2\Delta$His-^{3}D-Tic]	**11**	2235.40	62%	6.0
**6b**	Val-Ala-PABC	Dau	5.0	5.20	**I**	[^{2}His-^{3}Trp]	**12**	2313.47	70%	7.5
**6b**	Val-Ala-PABC	Dau	5.0	5.20	**II**	[$^2\Delta$His-^{3}D-Tic]	**13**	2149.31	65%	6.6
**9a**	Val-Cit-PABC	PTX	5.2	3.50	**I**	[^{2}His-^{3}Trp]	**14**	2840.10	84%	7.1
**9a**	Val-Cit-PABC	PTX	4.5	3.03	**II**	[$^2\Delta$His-^{3}D-Tic]	**15**	2675.66	77%	5.6
**9b**	Val-Ala-PABC	PTX	3.3	2.35	**I**	[^{2}His-^{3}Trp]	**16**	2754.01	80%	4.7
**9b**	Val-Ala-PABC	PTX	4.3	3.07	**II**	[$^2\Delta$His-^{3}D-Tic]	**17**	2589.84	56%	4.4
**20**	none	Dau	5.0	7.80	**I**	[^{2}His-^{3}Trp]	**23**	1994.120	52%	7.3
**20**	none	Dau	5.0	7.80	**II**	[$^2\Delta$His-^{3}D-Tic]	**24**	1829.955	50%	6.5
**22**	none	PTX	2.6	2.40	**I**	[^{2}His-^{3}Trp]	**25**	2434.652	53%	2.8
**22**	none	PTX	2.6	2.40	**II**	[$^2\Delta$His-^{3}D-Tic]	**26**	2270.487	65%	3.2

RP-HPLC profile and ESI-ion trap mass spectrum

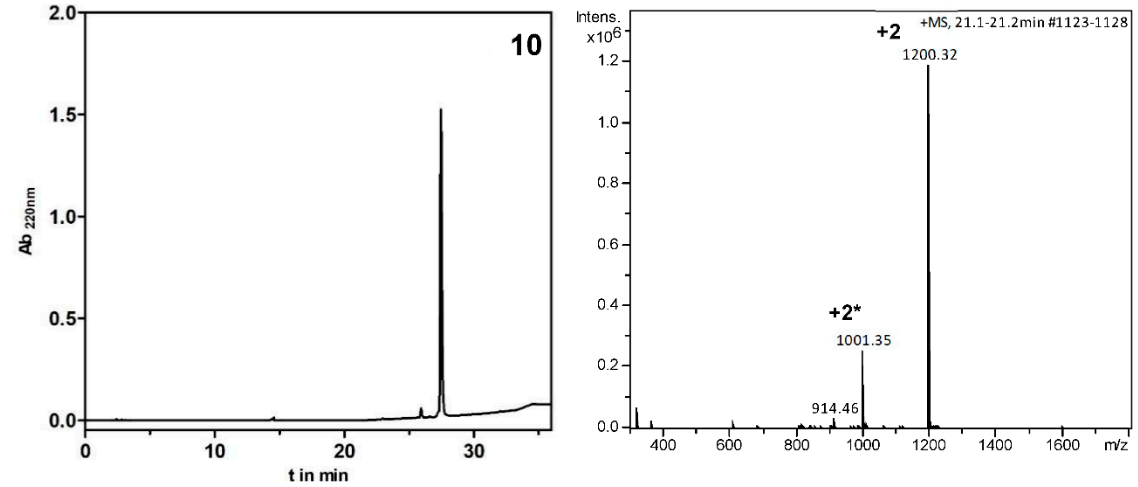

Figure S1. RP-HPLC profile and ESI-ion trap mass spectrum of GnRH-III-[2His-3Trp,8Lys(glutaryl-Val-Cit-PABC-Dau)] conjugate (**10**) (MW_{cal} /MW_{exp} = 2399.57/2398.63 g/mol).

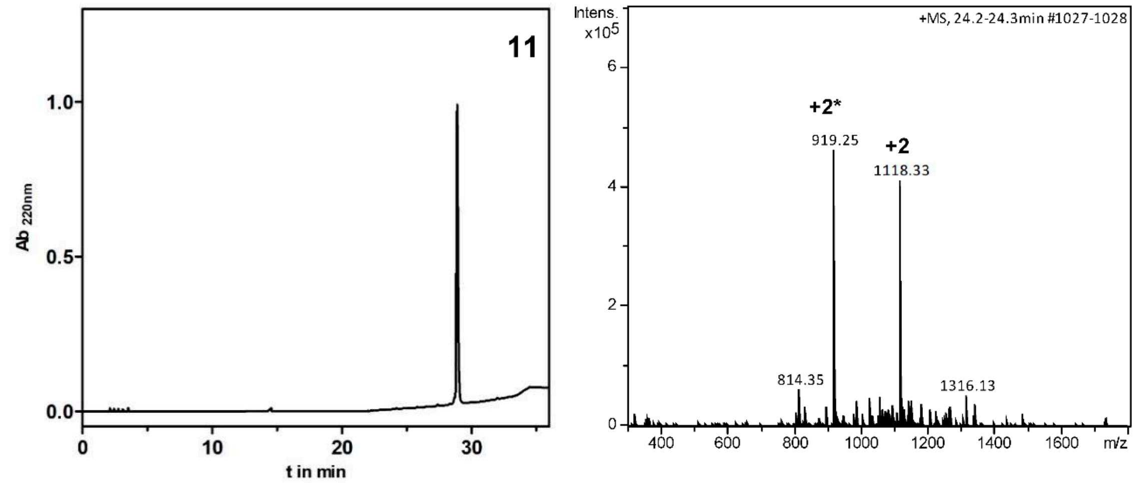

Figure S2. RP-HPLC profile and ESI-ion trap mass spectrum of GnRH-III-[2ΔHis-3D-Tic,8Lys(glutaryl-Val-Cit-PABC-Dau)] conjugate (**11**) (MW_{cal} /MW_{exp} = 2235.40/2234.45 g/mol).

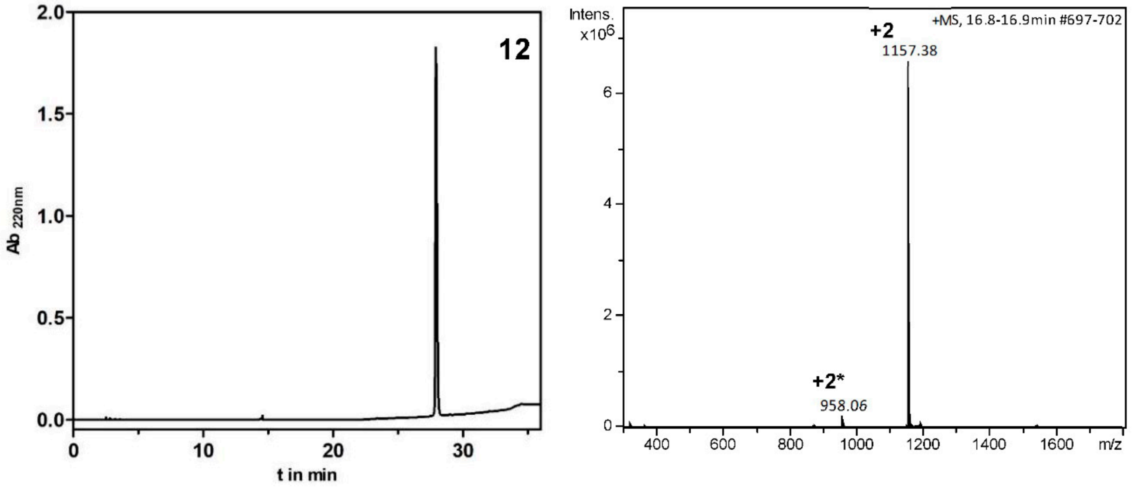

Figure S3. RP-HPLC profile and ESI-ion trap mass spectrum of GnRH-III-[2His-3Trp,8Lys(glutaryl-Val-Ala-PABC-Dau)] conjugate (**12**) (MW_{cal} /MW_{exp} = 2235.40/2234.45 g/mol).

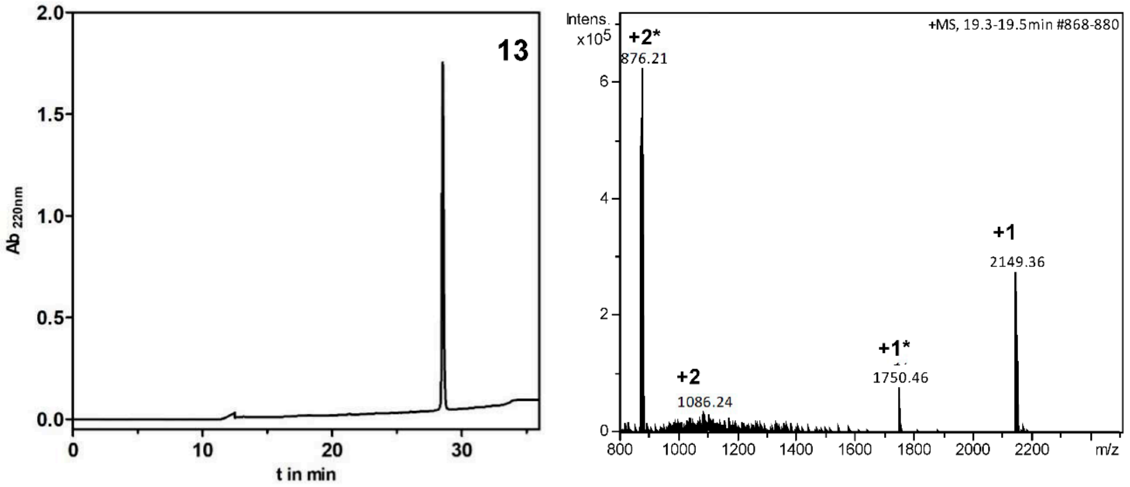

Figure S4. RP-HPLC profile and ESI-ion trap mass spectrum of GnRH-III-[$^{2}\Delta$His-^{3}D-Tic, ^{8}Lys(glutaryl-Val-Ala-PABC-Dau conjugate (**13**) (MW_{cal} /MW_{exp} = 2149.31/2148.35 g/mol).

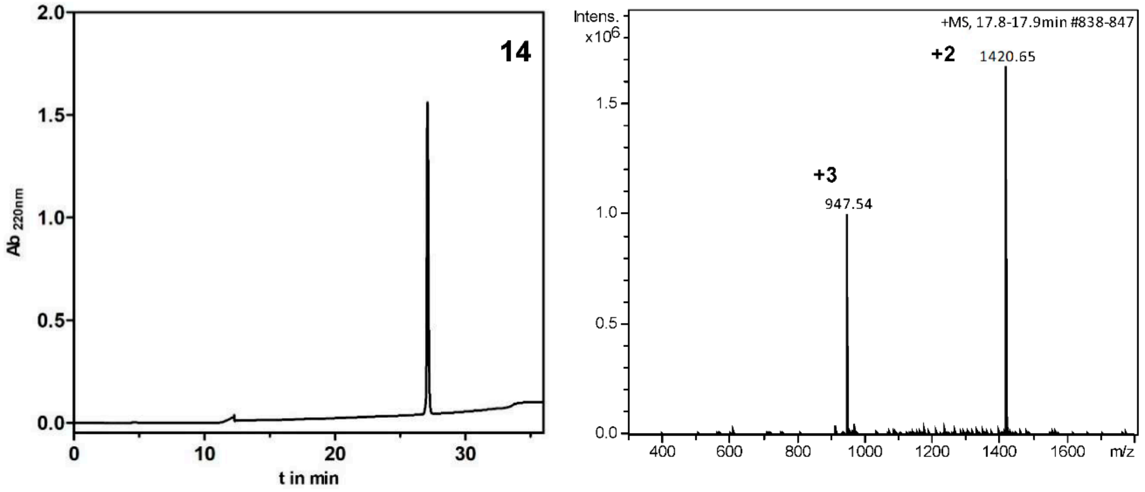

Figure S5. RP-HPLC profile and ESI-ion trap mass spectrum of GnRH-III-[^{2}His-^{3}Trp,^{8}Lys(glutaryl-Val-Cit-PABC-diamine-PTX)] conjugate (**14**) (MW_{cal} /MW_{exp} = 2840.10/2839.30 g/mol).

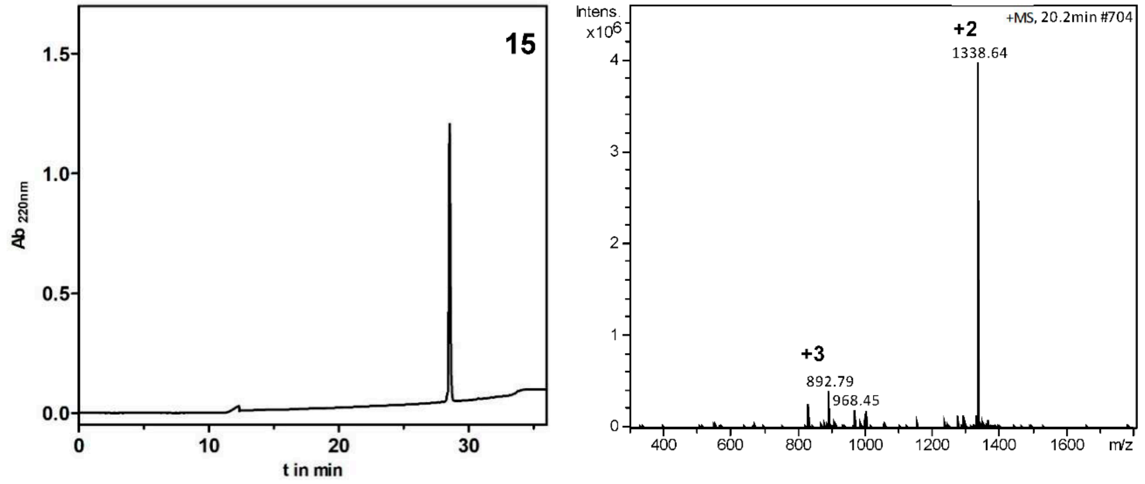

Figure S6. RP-HPLC profile and ESI-ion trap mass spectrum of GnRH-III-[$^{2}\Delta$His-^{3}D-Tic,^{8}Lys(glutaryl-Val-Cit-PABC-diamine-PTX)] conjugate (**15**) (MW_{cal} /MW_{exp} = 2675.66/2675.28 g/mol).

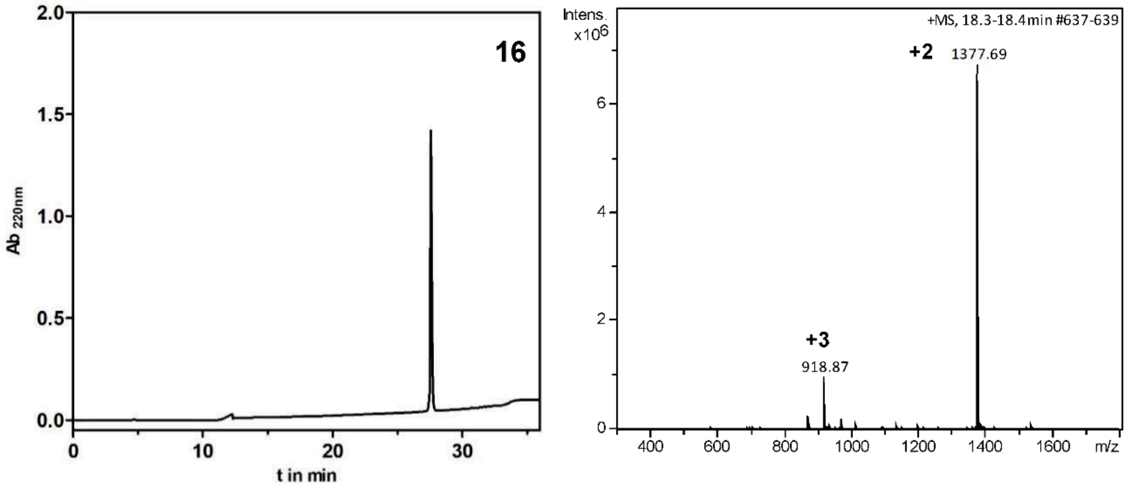

Figure S7. RP-HPLC profile and ESI-ion trap mass spectrum of GnRH-III-[^{2}His-^{3}Trp,^{8}Lys(glutaryl-Val-Ala-PABC-diamine-PTX)] conjugate (**16**) (MW_{cal} /MW_{exp} = 2754.01/2753.38 g/mol).

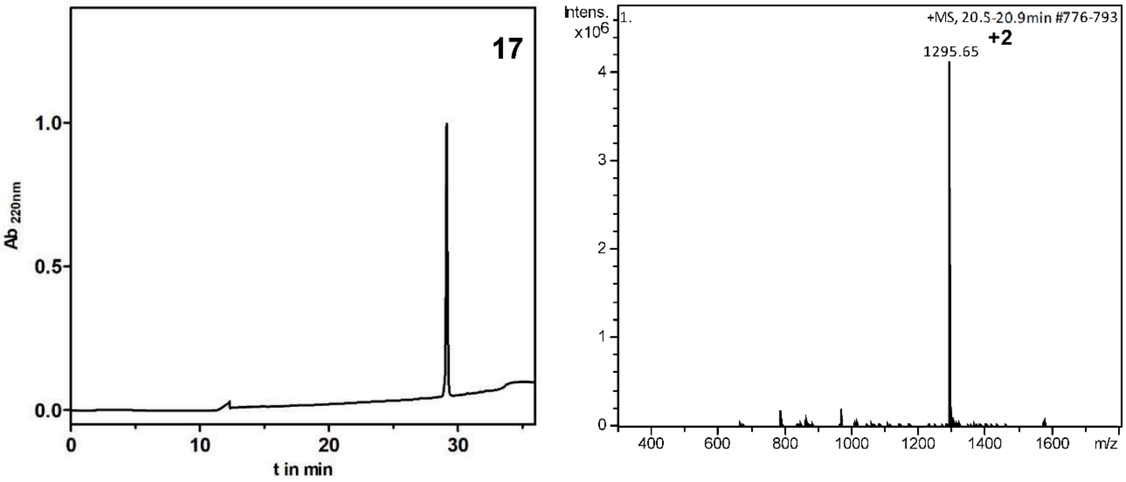

Figure S8. RP-HPLC profile and ESI-ion trap mass spectrum of GnRH-III-[^{2}ΔHis-^{3}D-Tic, ^{8}Lys(glutaryl-Val-Ala-PABC-diamine-PTX)] conjugate (**17**) (MW_{cal} /MW_{exp} = 2589.84/2589.30 g/mol).

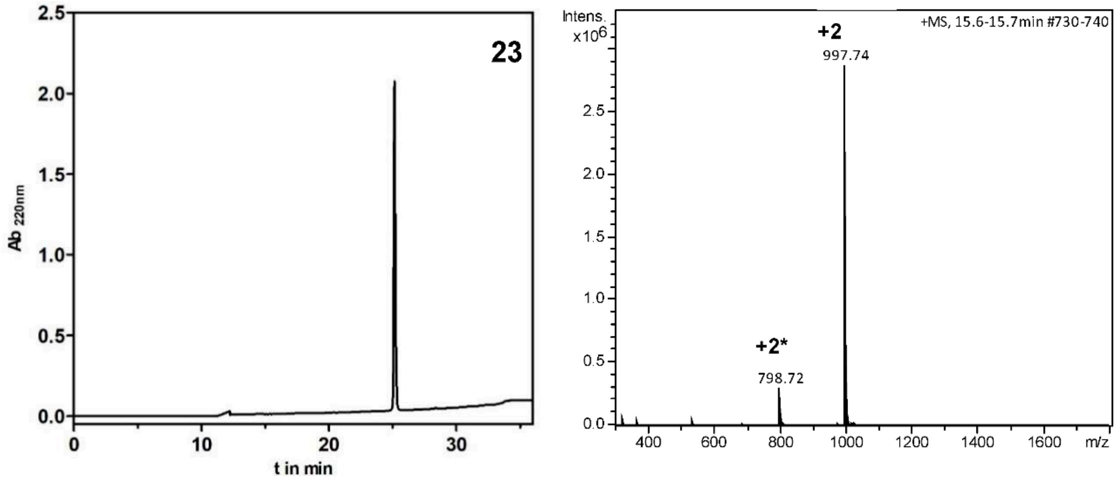

Figure S9. RP-HPLC profile and ESI-ion trap mass spectrum of GnRH-III-[^{2}His-^{3}Trp,^{8}Lys(glutaryl-Dau)] conjugate (**23**) (MW_{cal} /MW_{exp} = 1994.12/1993.48 g/mol).

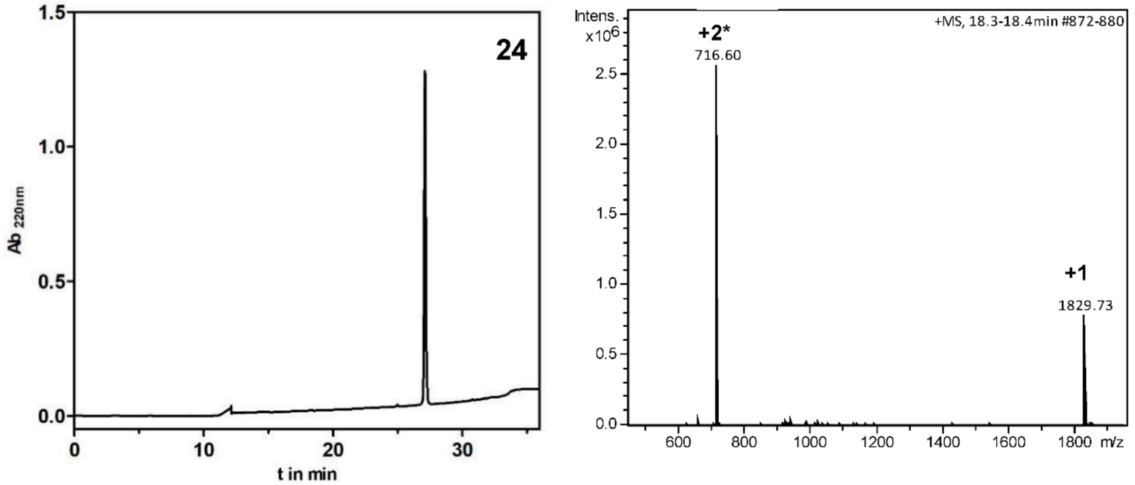

Figure S10. RP-HPLC profile and ESI-ion trap mass spectrum of GnRH-III-[[2]ΔHis-[3]D-Tic,[8]Lys(glutaryl-Dau)] conjugate (**24**) (MW_{cal} /MW_{exp} = 1829.95/1829.75 g/mol).

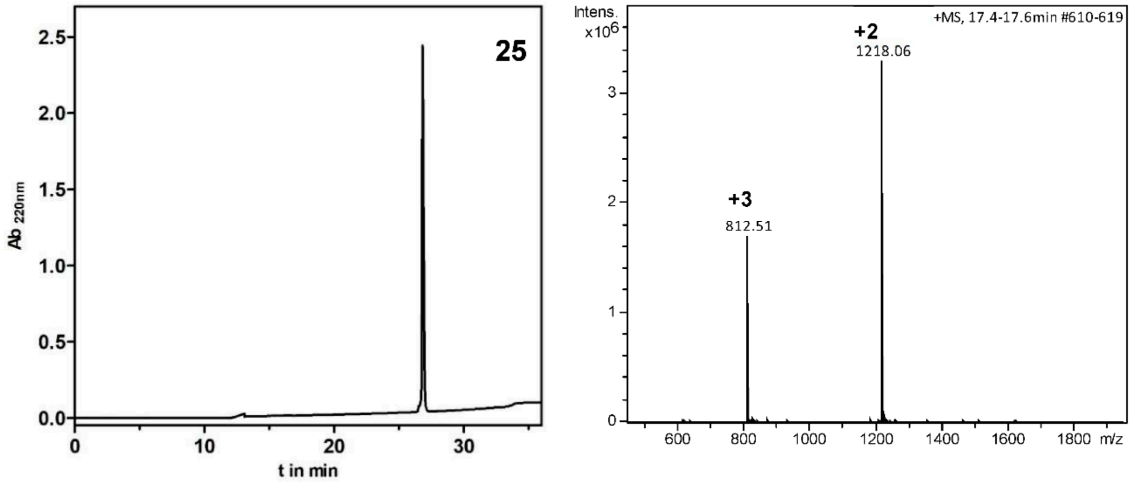

Figure S11. RP-HPLC profile and ESI-ion trap mass spectrum of GnRH-III-[[2]His-[3]Trp,[8]Lys(glutaryl-diamine-PTX)] conjugate (**25**) (MW_{cal} /MW_{exp} = 2434.65/2434.12 g/mol).

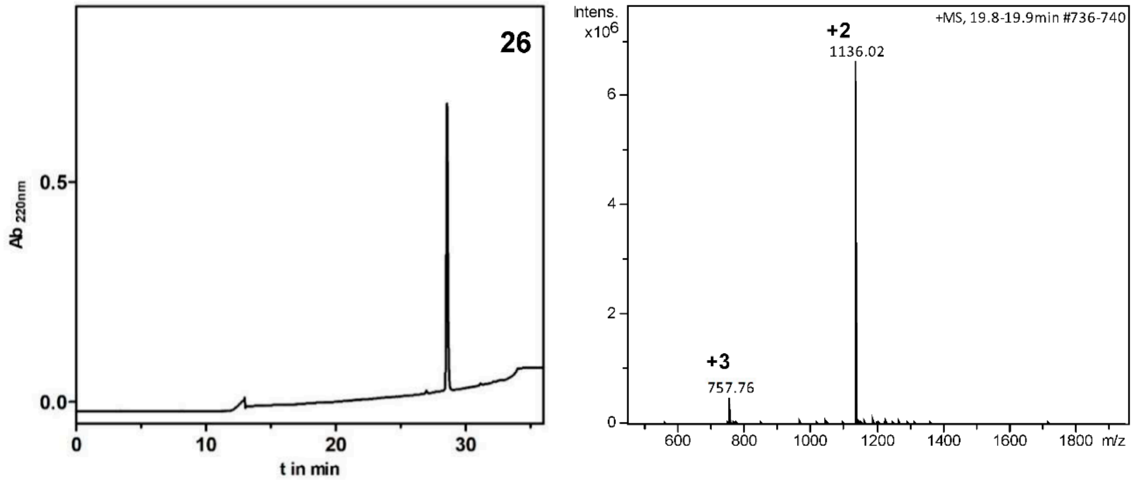

Figure S12. RP-HPLC profile and ESI-ion trap mass spectrum of GnRH-III-[[2]ΔHis-[3]D-Tic, [8]Lys(glutaryl-diamine-PTX)] conjugate (**26**) (MW_{cal} /MW_{exp} = 2270.49/2270.04 g/mol).

Table S2: Chemical characteristics of the cleavable and non-cleavable GnRH-III-Dau and -PTX bioconjugates

Drug	Code	GnRH-III-[^{4}Lys(Bu), ^{8}Lys(linker-drug)]	Linker	Purity [%]	RP-HPLC R_t [min][a]	MW_{cal}/MW_{exp} [g/mol][b]
Dau	**10**	[^{2}His-^{3}Trp]	Val-Cit	≥97	27.45	2399.57/2398.63
	**11**	[^{2}ΔHis-^{3}D-Tic]	Val-Cit	≥98	28.88	2235.40/2234.45
	**12**	[^{2}His-^{3}Trp]	Val-Ala	>99	27.92	2313.47/2312.74
	**13**	[^{2}ΔHis-^{3}D-Tic]	Val-Ala	≥98	28.53	2149.31/2148.35
	**23**	[^{2}His-^{3}Trp]	non-cleava-ble	≥99	25.15	1994.12/1993.49
	**24**	[^{2}ΔHis-^{3}D-Tic]	non-cleava-ble	≥98	27.10	1829.95/1829.75
PTX	**14**	[^{2}His-^{3}Trp]	Val-Cit	≥99	27.10	2840.10/2839.30
	**15**	[^{2}ΔHis-^{3}D-Tic]	Val-Cit	≥98	28.53	2675.66/2675.28
	**16**	[^{2}His-^{3}Trp]	Val-Ala	≥98	27.58	2754.01/2753.38
	**17**	[^{2}ΔHis-^{3}D-Tic]	Val-Ala	≥97	29.13	2589.84/2589.30
	**25**	[^{2}His-^{3}Trp]	non-cleava-ble	>98	26.83	2434.65/2434.11
	**26**	[^{2}ΔHis-^{3}D-Tic]	non-cleava-ble	≥99	28.57	2270.49/2270.04

[a]Column: Macherey-Nagel Nucleosil C18 column (250 mm x 4.6 mm) with 5 µm silica (100 Å pore size); gradient: 0 min 0% B, 5 min 0% B, 30 min 90% B; eluents: 0.1% TFA in water (A) and 0.1% TFA in acetonitrile-water (80:20, v/v) (B); flow rate: 1 mL/min; detection at 220 nm. [b]Bruker Daltonics Esquire 3000+ ion trap mass spectrometer.

Western blot analysis of A2780 and Panc-1 cancer cells

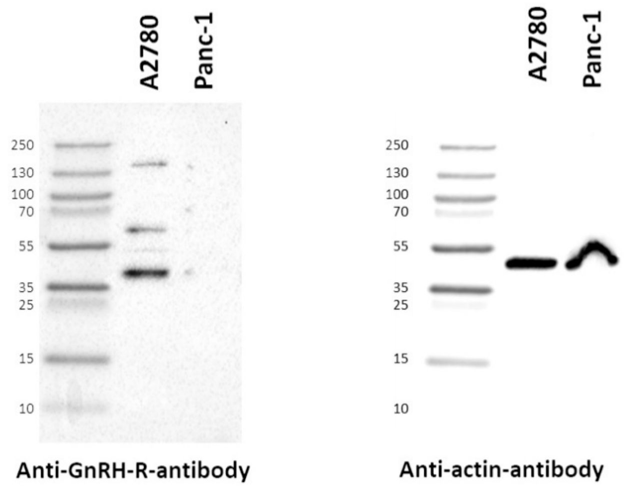

Figure S13. Western blot performed on whole cell lysates of A2780 and Panc-1 cancer cells. Anti-GnRH-R antibody (Proteintech, 19950-1AP) (left). Actin expression was evaluated as loading control (Santa Cruz Biotechnology, sc-1616 (right). Band at 38 kDa represents the full length human GnRH-R; the signals at higher molecular weight (55-70 kDa) are assumed to be glycosylated forms of the GnRH-R.

Lysosomal degradation of in presence of rat liver lysosomal homogenate

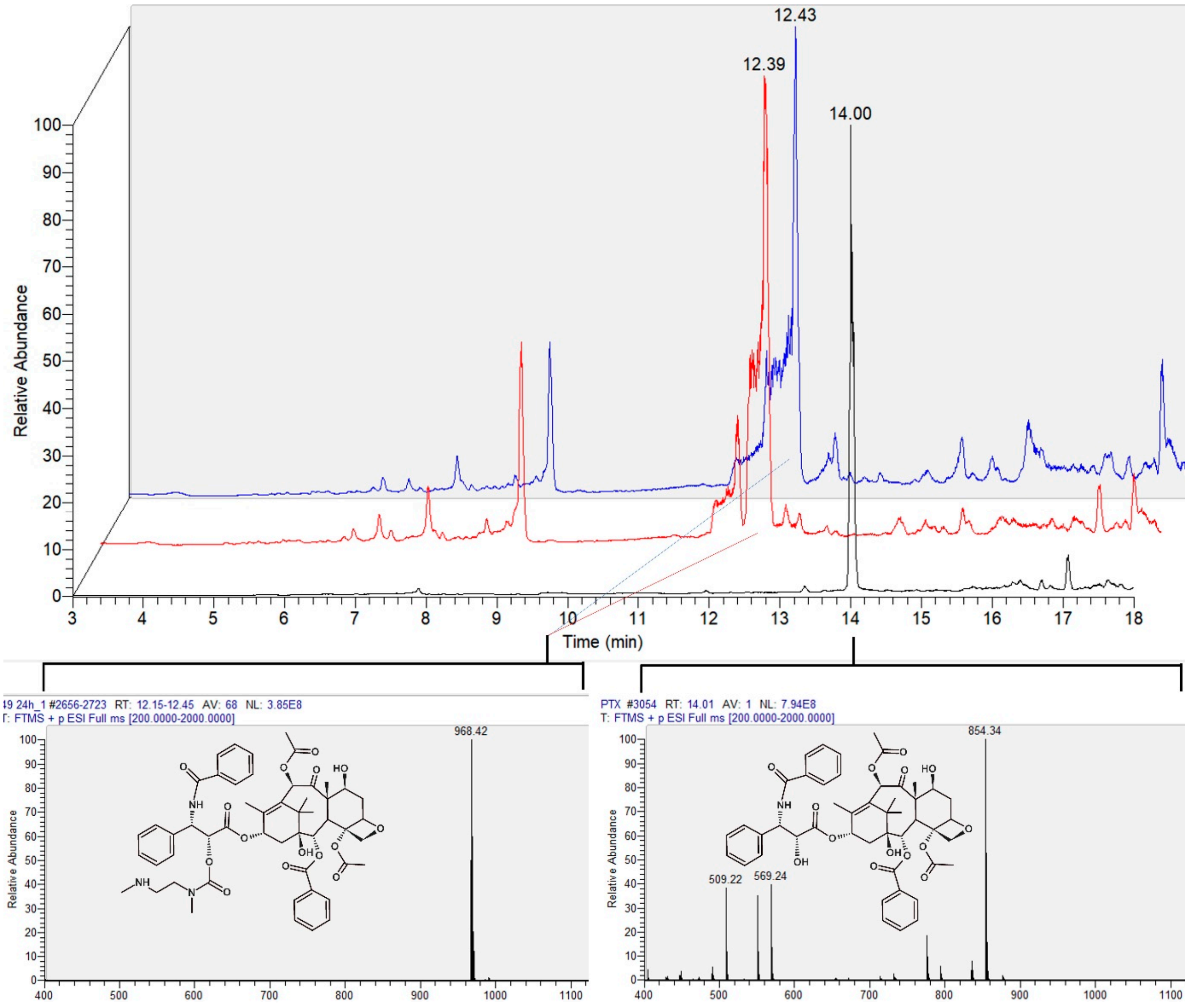

Figure S14. Degradation of the GnRH-III-PTX conjugates (**14**, **16**) in presence of lysosomal rat liver homogenate. LC chromatograms of PTX (black), **14** (red) and **16** (blue) after 24 hours degradation and MS spectra the released PTX-prodrug and PTX.
